# Activated dendritic cells delivered in tissue compatible biomatrices induce *in-situ* anti-tumor CTL responses leading to tumor regression

**DOI:** 10.18632/oncotarget.9529

**Published:** 2016-05-20

**Authors:** Vivek Verma, Young Kim, Min-Cheol Lee, Jae-Tae Lee, Sunghoon Cho, In-Kyu Park, Jung Joon Min, Je Jung Lee, Shee Eun Lee, Joon Haeng Rhee

**Affiliations:** ^1^ Clinical Vaccine R&D Center, Chonnam National University Medical School, Gwangju, South Korea; ^2^ Department of Pathology, Chonnam National University Medical School, Gwangju, South Korea; ^3^ Department of Nuclear Medicine, Kyungpook National University School of Medicine, Daegu, South Korea; ^4^ School of Mechanical Systems Engineering, Chonnam National University, Gwangju, South Korea; ^5^ Department of Biomedical Science, Chonnam National University Medical School, Gwangju, South Korea; ^6^ Department of Nuclear Medicine, Chonnam National University Medical School, Gwangju, South Korea; ^7^ Research Center for Cancer Immunotherapy, Hwasun Hospital, Chonnam National University, Hwasun, South Korea; ^8^ Department of Pharmacology and Dental Therapeutics, School of Dentistry, Chonnam National University, Gwangju, South Korea; ^9^ Department of Microbiology, Chonnam National University Medical School, Gwangju, South Korea; ^10^ Present address: GRU Cancer Center, GRU, Augusta, GA, USA

**Keywords:** dendritic cells, tumor, immunotherapy, biomatrices

## Abstract

Dendritic cell (DC) based anti-cancer immunotherapy is well tolerated in patients with advanced cancers. However, the clinical responses seen after adoptive DC therapy have been suboptimal. Several factors including scarce DC numbers in tumors and immunosuppressive tumor microenvironments contribute to the inefficacy of DCs as cellular vaccines. Hence DC based vaccines can benefit from novel methods of cell delivery that would prevent the direct exposure of immune cells to suppressive tumor microenvironments. Here we evaluated the ability of DCs harbored in biocompatible scaffolds (referred to as biomatrix entrapped DCs; beDCs) in activating specific anti-tumor immune responses against primary and post-surgery secondary tumors. Using a preclinical cervical cancer and a melanoma model in mice, we show that single treatment of primary and post-surgery secondary tumors using beDCs resulted in significant tumor growth retardation while multiple inoculations were required to achieve a significant anti-tumor effect when DCs were given in free form. Additionally, we found that, compared to the tumor specific E6/E7 peptide vaccine, total tumor lysate induced higher expression of CD80 and CD40 on DCs that induced increased levels of IFNγ production upon interaction with host lymphocytes. Remarkably, a strong immunocyte infiltration into the host-implanted DC-scaffold was observed. Importantly, the host-implanted beDCs induced the anti-tumor immune responses in the absence of any stromal cell support, and the biomatrix structure was eventually absorbed into the surrounding host tissue. Collectively, these data indicate that the scaffold-based DC delivery may provide an efficient and safe way of delivering cell-based vaccines for treatment of primary and post-surgery secondary tumors.

## INTRODUCTION

Cancers are a leading cause of human mortality worldwide [1]. Each year millions of cancer patients are treated using anticancer therapies such as chemotherapy, radiotherapy, surgery and immunotherapy. In particular, surgery is the most definitive anti-tumor measure against solid resectable tumors. However, due to strong immunosuppressive environment of post-surgery secondary tumors [2], tumor relapse in more than 40% of treated patients compromises the therapeutic efficacy of surgery [3]. Hence, immune-modulation using lymphodepletion to remove immune-suppressive cells or active immunization using cancer specific peptide or cell based vaccines has been suggested as an adjuvant treatment to surgery [4].

Dendritic cells (DCs) are antigen presenting cells (APCs) that work at the interface of innate and adaptive immunity. Vaccination strategies involving DCs have been developed to induce tumor-specific T cells that can reduce the tumor mass and induce immunological memory to suppress tumor relapse. For antigen presentation, DCs can be activated by various means including antigen coupling via DC-specific antibodies or tumor specific antigen capture under *in vivo* and/or *ex vivo* conditions [5]. Interestingly, *ex vivo* generated and activated DCs have been found to induce tumor specific T cell responses *in vivo*. However, DC-based vaccines are influenced negatively by the low trafficking into the tumor lesions [6] and by the suppressive tumor microenvironments [7]. In fact, inhibitory signals generated by tumor cells are sufficient to induce a transition of adoptively transferred cells from stimulatory to suppressive states facilitating the immune escape of tumor cells [8]. Hence, alternate modes of adoptive cell delivery that would prevent the exposure of the adoptively transferred cells to the tumor microenvironments while promoting efficient lymphocyte activation are required [9].

Lymph nodes (LNs) are secondary lymphoid organs (SLOs) that are located strategically to recruit naive lymphocytes from blood and antigen-carrying DCs from peripheral tissues [10]. In the classical model of lymphocyte activation, DCs in SLOs activate cognate T cells that, after proliferation, enter the blood circulation and get recruited to the tissues to react against relevant antigens [11]. However, a number of studies suggest that the classical paradigm of lymphocyte activation in SLOs may be overly simplistic as antigen-specific lymphocyte priming can also be achieved extranodally [12, 13], often times in conditionally established tertiary lymphoid structures (TLSs). This raises the possibility that activated DCs delivered in tissue compatible biomatrices may act as a site for immune cell activation. In light of these observations, in the present proof-of-concept study we tested if activated bone marrow derived DCs (BMDCs), delivered in tissue compatible scaffolds would result in generation of antitumor immune responses and subsequent tumor regression. Here we report the generation of anti-tumor immune responses by beDCs and their conversion into immune-organoids capable of inducing IFNγ-secreting activated lymphocytes.

## RESULTS

### Tumor associated antigen (TAA) induces stronger DC activation leading to higher IFNγ production upon interaction with lymphocytes

**Figure 1:**
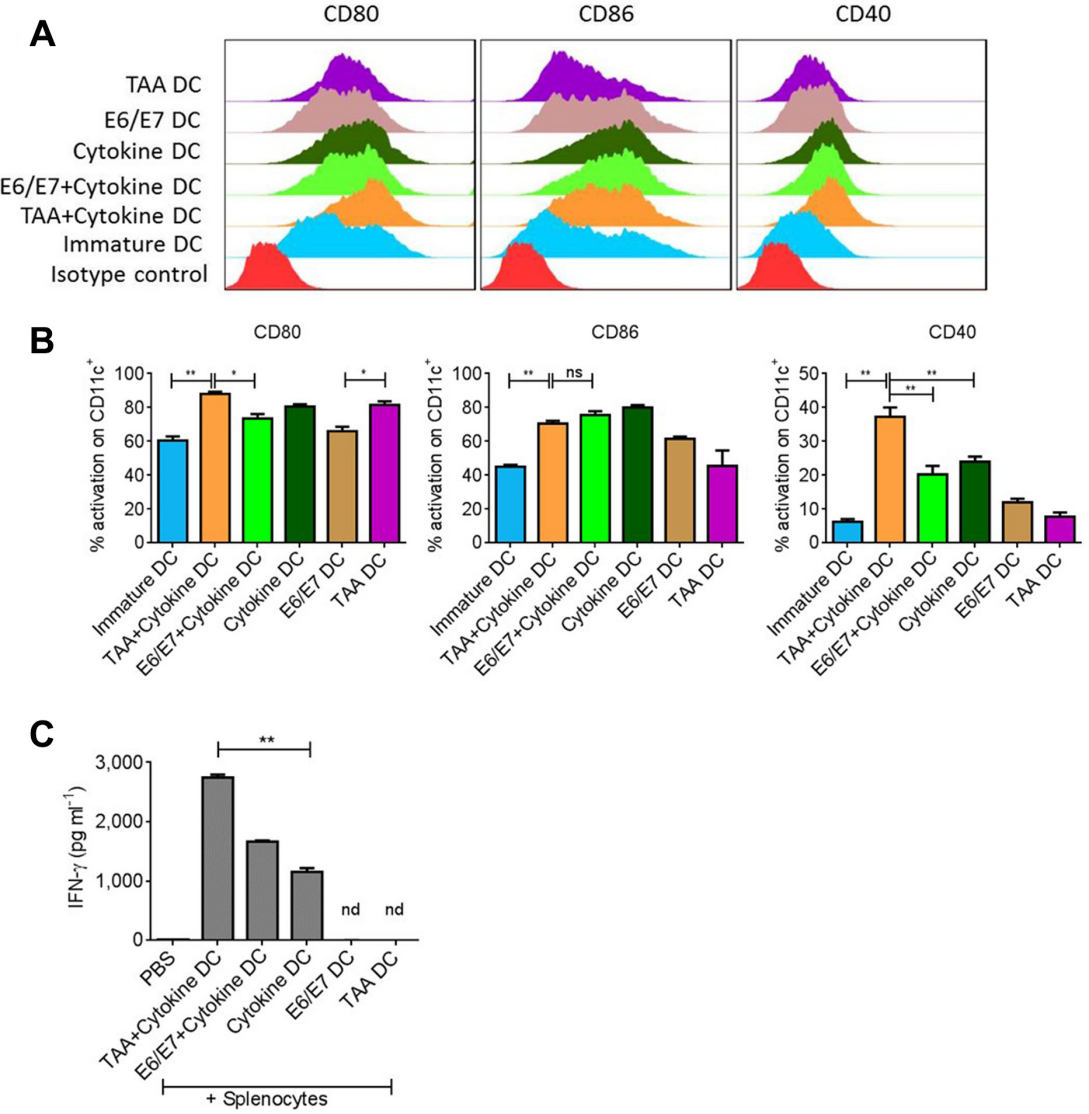
Total tumor lysate induces DC activation Bone marrow derived DCs were exposed to tumor associated antigen (TAA) or antigen specific E6/E7 peptides in presence or absence of proinflammatory cytokine cocktail. (**A**) Representative FACS analysis of costimulatory molecules on DCs after 18–24 hours of activation. (**B**) Statistical analysis of DC activation from A. (**C**) Levels of IFNγ induced after incubation of variously activated DCs with splenocytes for 3–4 days. Data are shown as + SEM. *p-value < 0.05, **p-value. < 0.01.

For appropriate anti-tumor responses, we first established the optimum conditions for DC activation. DCs were subjected to the tumor associated antigen (TAA) prepared from TC1 cell lysate or purified human papilloma virus (HPV) specific E6/E7 peptide epitopes, with or without pro-inflammatory cytokine cocktail. We observed that TAA alone significantly increased the expression of CD80 on CD11c^+^ DCs though the expression of CD86 and CD40 was not significantly different from E6/E7 peptide-treated DC group (Figure 1A–1B). Importantly, there was an increase in the expression of respective activation markers if antigen was captured by DCs in the presence of pro-inflammatory cytokine cocktail. However, yet again the BMDCs activated with TAA+cytokine showed significantly higher expression of CD80 and CD40 than the BMDCs activated with E6/E7+cytokine (Figure 1A–1B). The levels of MHCII expression were insignificantly different in various groups (data not shown). Moreover, in line with CD40 expression on variously activated DCs, it was observed that DCs activated with TAA+cytokine induced significantly higher INFγ production upon interaction with splenocytes than the DCs from other activation groups (Figure 1C) (*P* < 0.01).

### Generation of fibrin-derived scaffolds for DC entrapment and tumor treatment

Next, we tested the potential of variously activated DCs to suppress the growth of primary (1^°^) tumors. For this, DC scaffolds were generated by incorporating activated DCs into the polymerized fibrin gels (Supplementary Figure S1). Fibrinogen is a glycoprotein approved by US Food and Drug Administration that, in the presence of thrombin, forms moldable biocompatible and biodegradable macroporous gel. The porosity and the rigidity of fibrin gels could be controlled by the initial concentration of fibrinogen protein and the units of thrombin used for gelification of the fibrinogen protein. Of note, the shape of final DC carrying scaffold could also be controlled. For example, for primary tumor treatment, we generated round scaffolds (Supplementary Figure S1A), whereas flat scaffolds were used for the treatment of post-surgery secondary tumors that would not interfere with tumor bulging (for ease of tumor measurement) (Supplementary Figure S1B).

**Figure 2:**
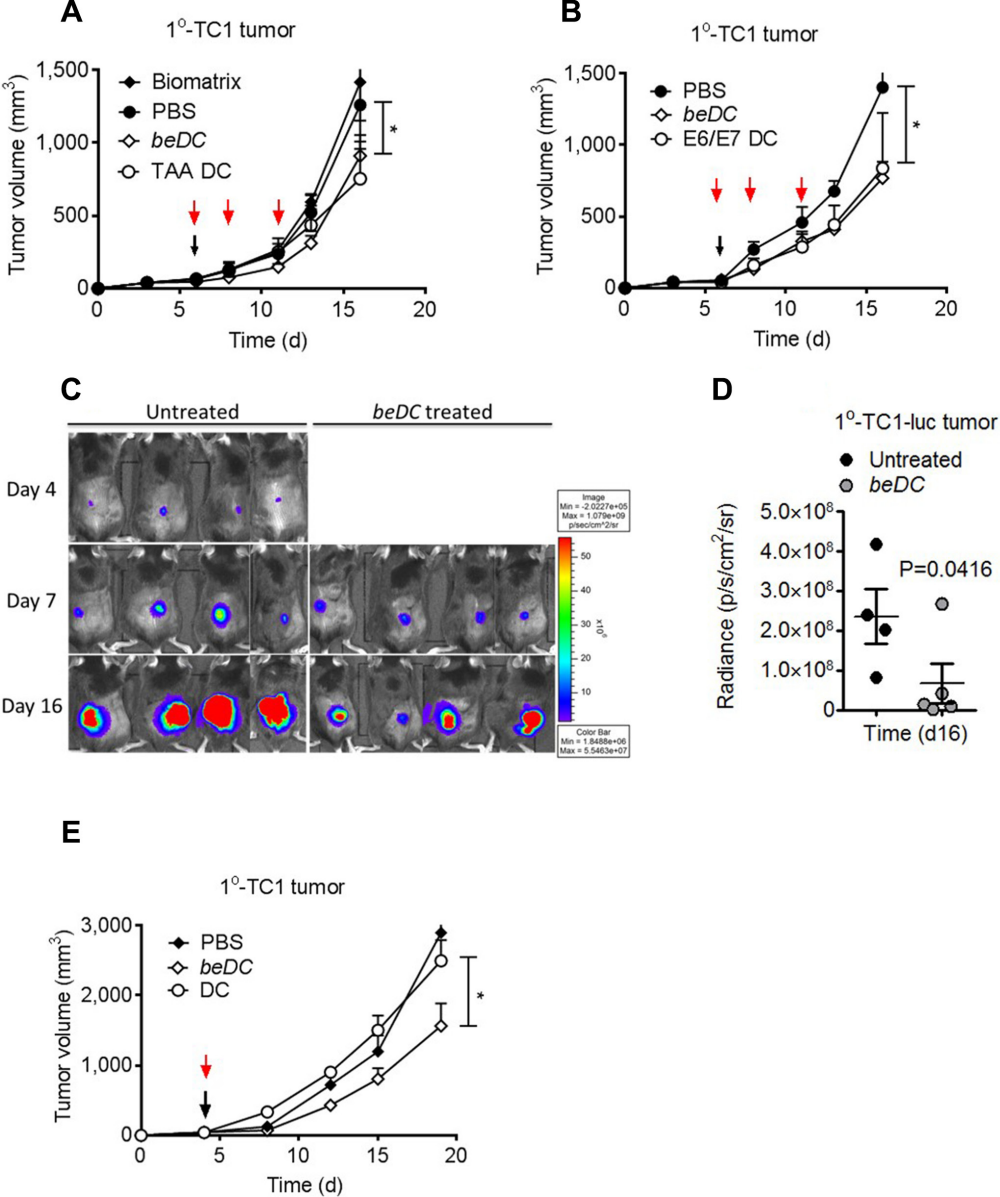
Treatment of TC1 primary (1°) tumors using DCs-in-scaffolds (beDC) or free DCs Mice bearing TC1 or TC1-luciferase (luc) cell induced 1° tumors were treated with free DCs or DC-in-scaffolds (beDC). (**A**) TAA-activated DCs were harbored in the fibrin scaffold and placed near the tumor site at day 5–6 (black arrow) or were inoculated in free form subcutaneously at three occasions (red arrow). (**B**) DCs were activated with E6/E7 peptides and inoculated into tumor-bearing mice either in free form (red arrows) or after harboring in fibrin scaffolds. (**C** and **D**) Luciferase expression of TC1-luc tumors after treatment by TAA-activated DCs given in fibrin scaffolds (beDC). (**E**) Comparison of treatment efficacy of single inoculation of scaffold harbored DCs (beDC) or free DCs in TC1 induced 1^°^ tumors. Error bars represent ± SEM. *p-value < 0.05.

Tumors were induced in C57BL/6 mice by subcutaneous (s.c.) inoculation of TC-1, TC-1-luciferase (luc) or B16 melanoma cells (5 × 10^5^/mouse) in the flank. Immunotherapy of the primary tumor was started when tumor volume was approximately 500 mm^3^. We observed that the treatment of primary tumors by a single implantation of beDCs (TAA or E6/E7 activated) resulted in a significant tumor growth retardation (*P* < 0.05) (Figure 2A–2D). However, to achieve similar anti-tumor effects using free-DCs multiple injections [14] were required (Figure 2A–2B) and a single inoculation of free-DCs was ineffective in inducing anti-tumor activity (Figure 2E). Interestingly, the anti-tumor effects of beDCs were dependent upon cell number as a scaffold carrying 1 × 10^5^ DCs arrested the tumor growth only during the earlier stages (Supplementary Figure S2A) and prolonged tumor suppression was achieved when DC number was increased to 1 × 10^6^ DCs (Figure 2A). Similar results were also noted against B16 melanoma wherein TAA-activated DC scaffolds resulted in significant tumor suppression (Supplementary Figure S2B). Since TAA+cytokine-activated DCs induced highest IFNγ upon interaction with host splenocytes (Figure 1C), in later experiments DCs (1 × 10^6^/scaffold) activated with TAA+cytokine were used for the tumor treatment. Taken together, these results highlighted that activated DCs delivered in a tissue compatible biomatrix resulted in significant tumor growth retardation.

### Antitumor effects elicited by beDCs are fortified by adjunct anti-cancer surgery

**Figure 3:**
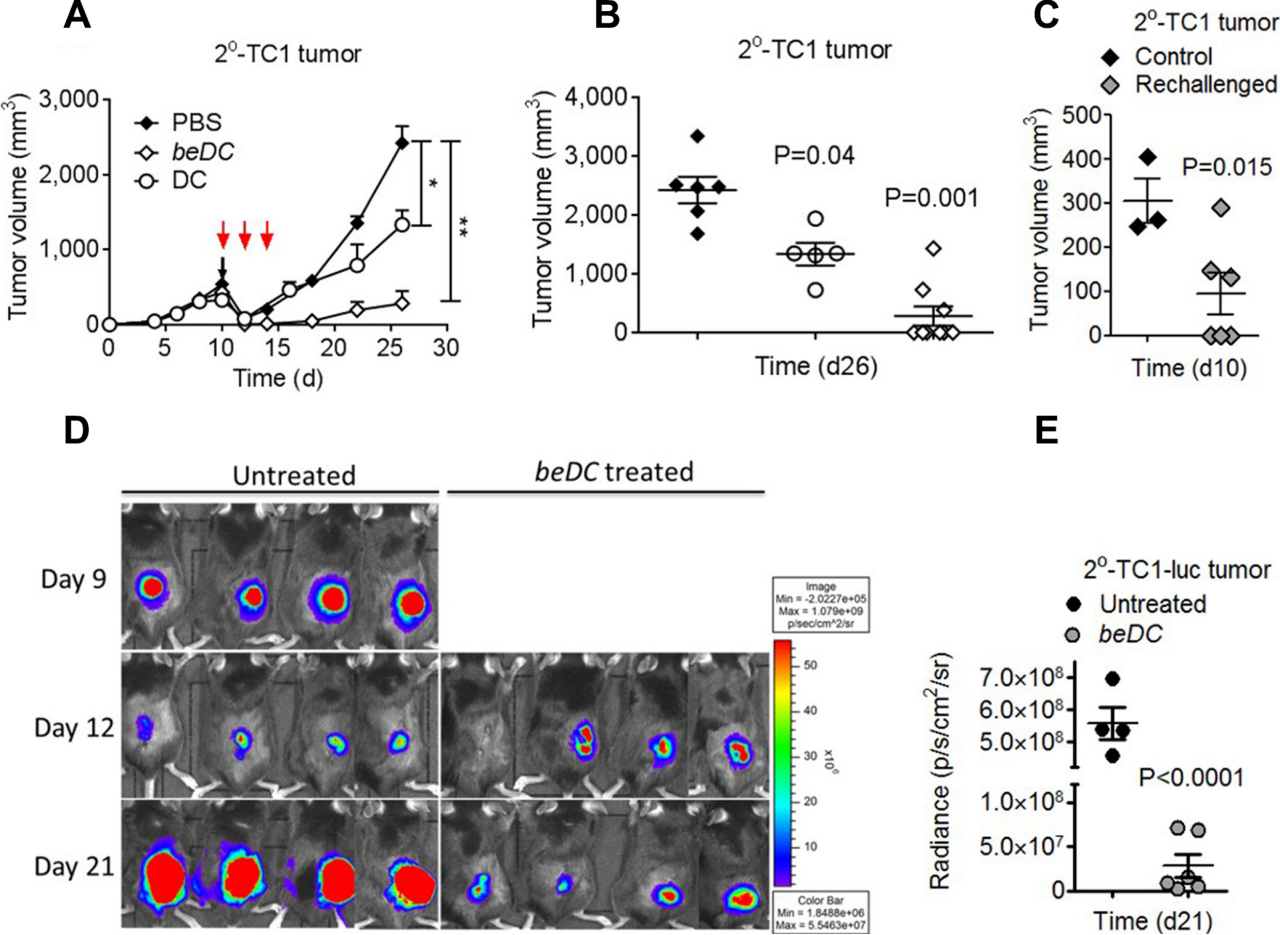
Induction and treatment of TC1 post-surgery secondary (2°) tumors using TAA activated DCs TC1 primary tumors were resected at day 10 when the tumor became 500 mm^3^ in size. (**A**) Resected tumors were treated with three injections of TAA-activated DCs (red arrows) or single placement of beDCs. (**B**) Statistical analysis of tumor volume at day 26. (**C**) Mice with completely regressed tumors in be DC group in B were re-challenged with TC1 cells and tumor growth was compared with the control group at day 10. Luciferase expression (**D**) and quantification (**E**) in 2° tumor-bearing mice after the DC scaffold treatment. Data is represented as ± SEM. *p-value < 0.05, **p-value < 0.01.

To explore the potential of DC scaffolds in eradicating post-surgery secondary tumors, TC1 or B16 solid tumors were partially resected and the beDCs were placed into the cavity left after the tumor resection (Supplementary Figure S1B2–S1B4). As a control, similar numbers of free DCs were inoculated s.c. near the tumor resection site three times on alternate days (Figure 3A). It was observed that, in the untreated group, the resected tumor grew back with increased rates and only modest tumor growth retardation was observed upon treatment with free DCs (*P* = 0.047) (Figure 3A–3B). In contrast, the treatment using beDCs resulted in a very significant tumor suppression (*P* < 0.001) (Figure 3A–3B, 3D–3E). Importantly, placement of DC scaffolds into the tumor resection site resulted in complete remission (CR) in > 65% of animal subjects and a significantly retarded tumor growth in the remainder of the animals (Figure 3B). The immune response induced by DC scaffold was found to be long lasting and systemic in nature because the 50% of long-term surviving mice (> 60 days) with complete tumor regression were able to resist a re-challenge with the same TC-1 tumor cells placed in the opposite flank, while animals in the control group showed a robust tumor growth post-inoculation (*n* = 3; age- and sex-matched) (Figure 3C). Moreover, significant inflammatory reactions were observed at the site of tumor cell injection in re-challenged CR animals (data not shown). Also, we observed a similar immune protection against B16 melanoma in mice (Supplementary Figure S2C), which highlighted the broad spectrum of anti-tumor effects of beDCs. Taken together, these results indicate that the delivery of DCs in biocompatible biomatrices is an effective mean to suppress the growth of primary as well as post-surgery residual tumors.

### Host immune cells infiltrate and interact with implanted DCs-in-scaffolds

**Figure 4:**
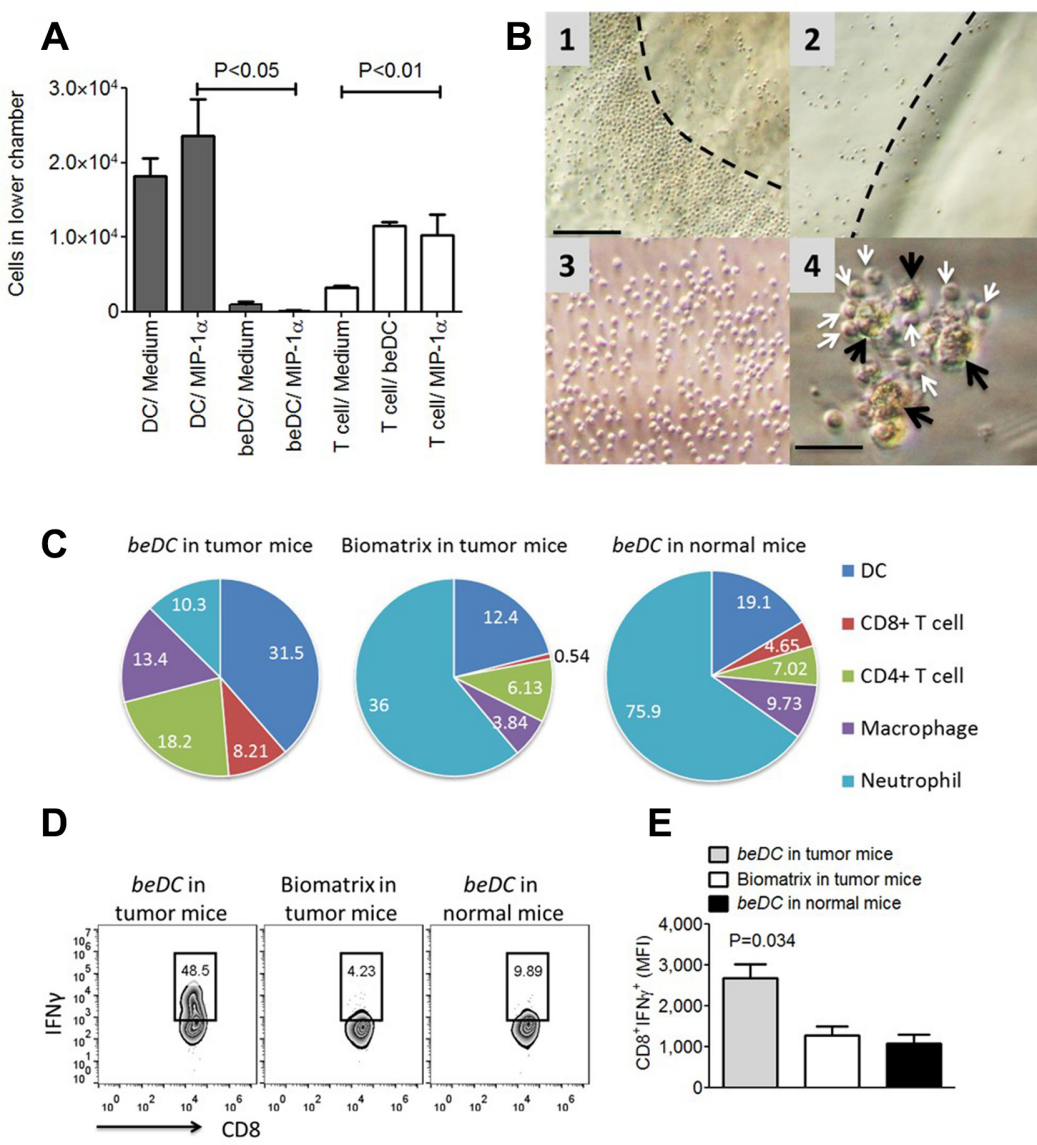
DCs in fibrin scaffolds facilitate immune cell movement *ex vivo* and *in vivo* (**A**) Migration profiles of DCs harbored in scaffolds (shaded bars) and lymphocytes (white bars) towards chemotactic signals in a transwell assay system. (**B**) Lymphocytic movement towards DCs in scaffolds under *ex vivo* conditions. Panel 1 shows the lymphocyte arrangement around the DC scaffold while 2 and 3 respectively shows the lymphocytic arrangement around biomatrix without DCs or in the absence of any stimulus. Dotted lines in 2 and 3 depicts the edge of biomatrix. Panel 4 shows the close association of infiltrated lymphocytes (white arrows) with biomatrix entrapped DCs (beDCs) (black arrows). Bar in 1 is equivalent to 100 μm and in 4 is equivalent to 25 μm. (**C**) Pie charts showing the absolute cell numbers (%) inside biomatrix harvested from various groups as shown in the figure. *n* = 3. (**D**) Representative FACS plots showing MFI and the statistical analysis of the plots (**E**) of the IFNγ induced upon activation of splenocytes harvested from various groups as shown in the figure.

Using a trans-well assay system, we first estimated the migration profile of DCs harbored inside the scaffold. For this, free DCs or beDCs were placed in the upper chamber and allowed to move towards MIP1α, a chemoattractant for DCs [15], in the lower chamber. We observed that free DCs moved robustly towards the chemoattractant, while DCs captured inside the biomatrix moved out significantly less towards the MIP1α signal (*P* < 0.05) (Figure 4A, shaded bars) indicating that scaffolds were holding the DCs avidly. Next, we estimated the movement of lymphocytes in response to the signals emanated from DCs inside the scaffold or MIP1α. For this assay, beDC or MIP1α was placed in the lower chamber and lymphocytes were placed in the upper chamber of transwell chamber. It was observed that the lymphocytes moved in significantly higher numbers (*P* < 0.01) towards beDCs as well as MIP1α in the lower chamber (Figure 4A, open bars). Interestingly, the lymphocytes that moved in response to beDCs gathered in an orderly fashion around the biomatrices and made intimate contacts with the DCs in the scaffolds after infiltrating into the biomatrix (Figure 4B1–4B4). Importantly, such orderly lymphocyte gathering was not detected around scaffolds without DCs (Figure 4B2) or due to self-aggregation (Figure 4B3).

Next we determined the fate of DC scaffolds when implanted into mouse body. For this, DC scaffolds were placed in either normal or tumor bearing mice. Also scaffold-only without DCs were placed in tumor bearing mice as a control. Biomatrices from various treatment groups were harvested 2–3 weeks post-implantation and processed to be analyzed for cellular composition. The DC-harboring biomatrices from tumor bearing mice had higher numbers of CD11c^+^ DCs (35.2% + 3.75%; mean + SEM) compared to that from normal mice (18.05% + 1.05%) (Figure 4C). As there was a significant infiltration of host DCs into empty biomatrix when placed in tumor bearing mice (16.7% + 4.3%), the high DC numbers seen inside biomatrix harvested from tumor bearing mice seem to be the cumulative sum of adoptively placed DCs-in-scaffolds and host-derived DCs (Figure 4C). Not only DCs but also lymphocytes, in particular CD8^+^ T-cells, were observed in significantly higher numbers inside the DC-carrying matrices harvested from tumor bearing mice (9.1% + 0.8%) than that harvested from normal mice (4.3% + 0.3%) (Figure 4C), indicating that the tumor-exposed lymphocytes had higher propensity to move towards the signals originated from DCs-in-scaffold. On the other hand, biomatrices devoid of DCs, when placed in tumor bearing mouse, had negligible numbers of CD8^+^ lymphocytes infiltrated into them (0.7% + 0.2%) highlighting that lymphocyte movement was a specific response to signals originated from DCs inside the scaffolds (Figure 4C). Interestingly, CD8^+^ cells in the spleen of tumor bearing mice that received beDCs had higher production of IFNγ upon stimulation with phorbol myristate acetate (PMA) + ionomycin (Figure 4D–4E). Notably, a large number of Ly6G^+^ neutrophils appeared around DC-scaffold implanted in normal mice (70.4% + 5.45%; mean + SEM) or empty-biomatrix implanted into tumor bearing mice (48.5% + 12.5%) (Figure 4C and Supplementary Figure S5). However, the numbers of neutrophils gathered around DC scaffolds in tumor bearing mice were significantly less than the other two groups (11.65% + 1.35%) (Figure 4C).

### Host implanted DC-carrying biomatrices develop physio-histological characteristics and are biodegradable

**Figure 5:**
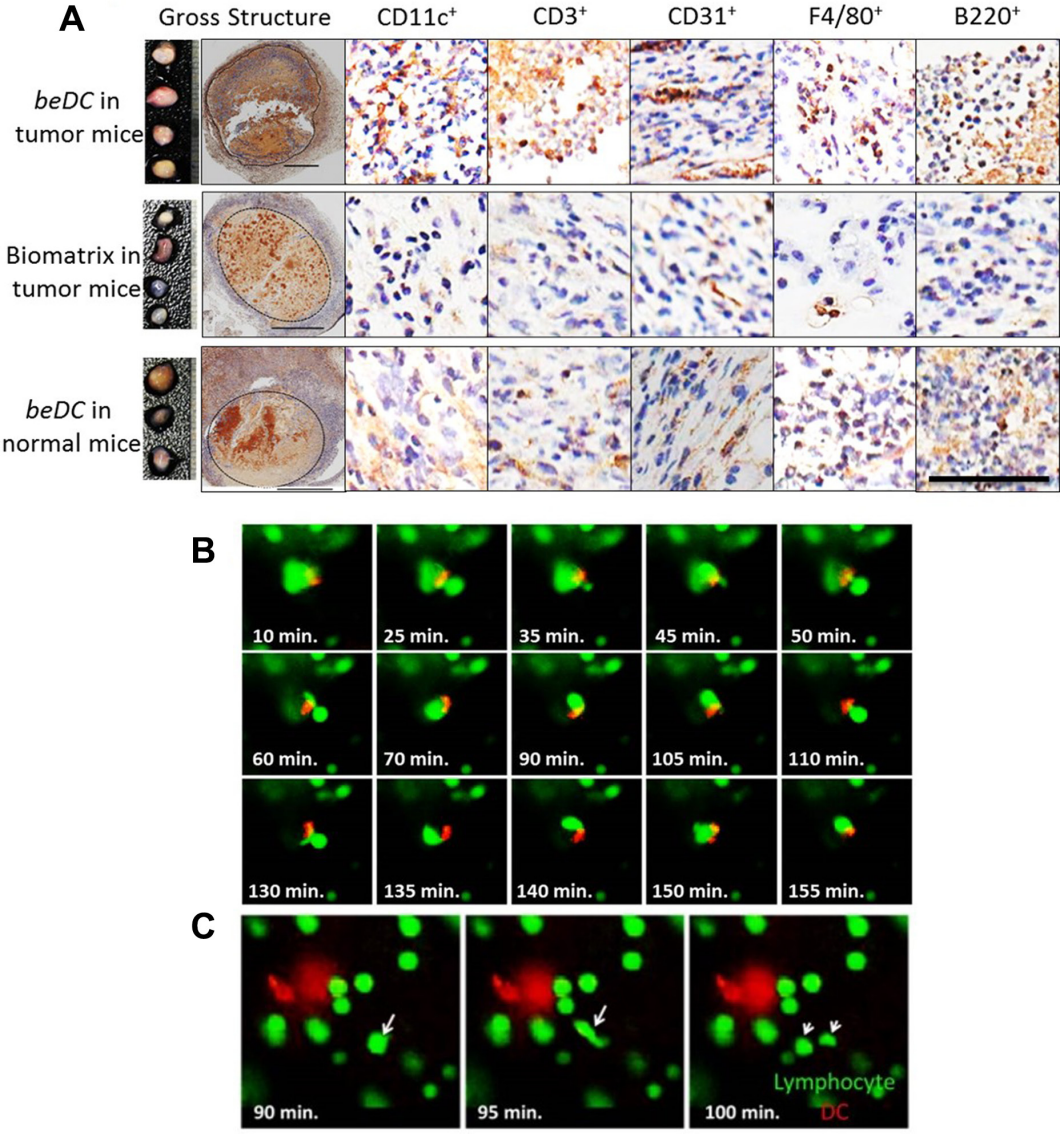
Lymphocyte interaction inside the DC harboring scaffolds DC scaffolds (beDC) or scaffolds only, placed in tumor bearing mice or DC-scaffold placed in normal mice were recovered two weeks post-implantation and processed for immunohistochemistry to demonstrate various cell types. (**A**) Relative size of the biomatrices harvested from various mice groups as depicted in the figure (far left panel). *n* = 3–5. The dotted line in ‘gross specimen’ differentiates the biomatrix from the reactive host-tissue. Representative images of biomatrix sections stained by the respective antibodies as indicated in the figure. The bar in the ‘gross structure’ is equivalent to 1 mm, whereas for other panels is equal to 100 μm. (**B**) Snapshots from Supplementary Movie S2 showing lymphocyte (green) movement and interaction with DCs (red) inside the biomatrix. The start point of the observation was set as the zero time point. (**C**) Lymphocyte division (white arrows) inside the biomatrix in the vicinity of DC (red). Representative snapshot from Supplementary Movie S3.

Further, the implanted DC scaffolds were harvested with tightly adhered reactive host tissue (Supplementary Figure S1A4) and analyzed by immunohistochemistry (IHC) (Figure 5A). Notably, in corroboration with the FACS data, high numbers of CD11c^+^ DCs were seen at the interface between biomatrix and host-reactive tissue (Figure 5A). Besides, high numbers of CD3^+^ lymphocytes, F4/80^+^ macrophages and B220^+^ cells were seen in the DC carrying scaffolds that were recovered from tumor bearing mice. We also observed a prominent induction of CD31^+^ vasculature (Figure 5A) in and around the DC-carrying matrices when placed in tumor bearing mice. Often times these vasculatures were seen to have lymphocytes associated with them, highlighting their functional nature. To evaluate the robustness of lymphocyte infiltration, we adoptively transferred CFSE^+^ lymphocytes intravenously into the DC scaffold-implanted tumor-bearing mice and tracked their movement into the biomatrices and other natural SLOs, 48 hrs post-infusion. Interestingly, we found that DC-carrying biomatrices at 2–3 weeks post-implantation had lymphocytes moved into them (Supplementary Figure S3A), which was with comparable propensities to the other natural secondary lymphoid organs [16] (Supplementary Figure S3B). Importantly, using live imaging system as described earlier [17], DCs in biomatrix were seen to interact actively with infiltrating host lymphocytes (Supplementary Movie S1). We observed that, though the DCs in scaffolds were stationary, the dendrites projected from them were making active physical contacts with infiltrating lymphocytes (Supplementary Movie S1). On the other hand, the infiltrating lymphocytes moved agilely inside the biomatrix and made intimate contacts with DCs for extended periods (Figure 5B and Supplementary Movie S2). Interestingly, under *in vitro* conditions, lymphocytes inside the biomatrix moved with a velocity (14.5 μm/sec + 1.92 μm/sec; mean + SEM) (Supplementary Figure S4) comparable to that under *in vivo* conditions [18] and often underwent cell division in the vicinity of DCs (Figure 5C and Supplementary Movie S3).

Finally, we observed that the structure of the empty biomatrix in tumor-bearing mice or the DC-carrying biomatrix in normal mice was significantly less durable (10–15 days) than that of the DC-carrying biomatrix placed in tumor-bearing mice (30–35 days) (Figure 5A and Supplementary Figure S5). Nonetheless, all engineered structures were biodegradable and were eventually absorbed into surrounding host tissues. Taken together, these results indicate that the tissue compatible biomatrices are effective tools to deliver activated DCs leading to an extra-nodal immune expansion and generation of protective anti-tumor immune responses.

## DISCUSSION

An emerging concern of the anti-tumor immunotherapy is the context in which effector cells are delivered into various immune compartments of the host [19]. Tumors have strong immunosuppressive microenvironments that tend to down-regulate the effector functions of adoptively transferred immune cells [20]. Moreover, the low frequency of transferred immune cells reaching the target tissue [21] and even the lower probability of meeting the minimum threshold for appropriate paracrine signaling [22] are potential hurdles to current cell-based antitumor immunotherapies. Hence, there are renewed interests in the scaffold-based immune cell delivery mechanisms [23, 24]. In the present study, we show that the delivery of DCs in biomatrices prevents the direct exposure of activated effector cells to immune-suppressive tumor microenvironments while inducing strong anti-tumor immune responses both locally and systemically. Biomatrices have been utilized for the delivery of vaccines and cells for tissue repair [25]. To best of our knowledge, this is the first time a biomatirx was used for the delivery of activated DCs for adjunctive immunotherapy of post-surgery secondary tumors.

In various diseases, tertiary lymphoid structures (TLSs) have been observed as sites for extranodal immune activation. In context to cancer prognosis, the generation of TLS is associated with a positive response to immune therapies [26], suggesting that the TLSs can be a site for generation of local anti-tumor immune responses. DCs are essential for TLS-mediated antitumor T cell responses highlighting the importance of DCs in extranodal immune activation [27]. However, for antitumor immune responses, DCs should be loaded with defined tumor antigens though in clinical practices such defined antigens are seldom available. The antigen preparations made from whole tumor lysate may harbor factors that would negatively affect the DC activation. However, in the present study, we observed that DCs could be programmed to induce effective anticancer T-cell immunity if the TAA was captured in a pro-inflammatory cytokin milieu. Such activated DCs express high levels of CD80, CD86 and CD40 and induce high amounts of IFNγ upon interaction with lymphocytes. Moreover, DCs activated with whole tumor lysates having multiple danger associated molecular patterns (DAMPs) have been reported to stimulate broader repertoire of T cells with multiple HLA types [28]. Contrary to this, DCs activated with tumor-specific peptide epitopes manifest shorter half-life [19] and have limited applicability in terms of human leukocyte antigen (HLA) restriction [28]. However, a caveat of using whole tumor lysate is the possibility of inducing autoimmunity against self-non-tumor antigens [29].

Several studies have noted suppressive microenvironments in post-surgery secondary tumors [2]. In the present study, we found that the conventional DC-based vaccine had only a limited anti-tumor effect that might be due to downregulation of the anti-tumor activity of immune cells by suppressive tumor microenvironments. However, beDCs were not directly exposed to the suppressive tumor environments. Moreover, the soluble factors secreted by the DCs in scaffolds were sufficient to attract and activate infiltrated host lymphocytes inside the biomatrix [33]. However, a lack of organized cellular clusters inside biomatrix may be due to the absence of specific soluble mediators that are required for a coordinate cellular organization [30]. Another important factor for the induction of robust immune responses in the DC-harboring biomatrix may be the biomaterial itself [32]. Fibrin is a physiological protein that elicits haptotactic and chemotactic responses in various cell types [34]. In particular, the fibrin degradation products (FDP) such as domain D are potent chemo-attractants for polymorphonuclear cells (PMNs). PMNs help the degradation of extraneous materials by elastase, proteinase 3 and cathespins stored in them [35], which may have resulted in profuse biomatrix digestion observed in DC scaffolds in normal mice or empty biomatrix in tumor mice (Supplementary Figure S5C–S5F). However, for *in vivo* sustenance of biomatrices, DC-derived signals as well as robust host lymphocyte movement seem to be important requisites as the durability of biomatrix was significantly shortened in the absence of any one of these factors. Nonetheless, eventual degradation of biomatrices in all groups showed self-limiting nature of biomatrix-mediated immune responses [36], a rather desirable phenomenon lacking in natural TLSs [37].

In summary, our results show that high numbers of appropriately activated DCs can be delivered effectively and made to stay for considerable periods in the tumor vicinity, resulting in the expansion of anti-tumor immune responses. However, this technology can further be benefited by incorporation of bioactive molecules such as specific drugs or cytokines that may have effects on tumors or the host immune system [24].

## MATERIALS AND METHODS

### Mice, antibodies and reagents

C57BL/6 mice were purchased from Charles River Laboratories and housed at the animal facility at Chonnam National University (CNU) Hwasun Hospital under specific pathogen free (SPF) conditions. All experiments were performed in accordance with the recommendations of the Institutional Animal Care and Use Committee (CNU IACUC-H-2012-33) at CNU, Gwangju, South Korea. Antibodies used for fluorescence-activated cell sorting (FACS) were fluorescein-, phycoerythrin (PE)-, PE-Cy5-, and allophycocyanin-labeled anti-CD11c (N418), anti-CD80 (B7-1), anti-CD86 (B7-2), anti-CD3 (17A2), anti-CD8α (53-6.7), anti-CD4 (GK1.5), anti-MHCII (M5/114.15.2), and anti-CD16/32 (clone 2.4G2). Anti-CD31 polyconal antibody, anti-CD3G (EPR4517), anti-CD11c (N418) and goat polyclonal anti-Armenian hamster IgG (Biotin) (all from abcam^®^), while anti-F4/80 (CI-A3-1) (Novus Biologicals) and anti-CD45R (B220) (RA3-6B2) (eBioscience) were used for immunohistochemistry (IHC) purpose. ELISAs for IFN-γ were performed using kits from BD Bioscience. Horseradish peroxidase (HRP)-conjugated goat anti-mouse IgG1 and alkaline phosphatase-conjugated anti-mouse IgM antibodies were from Dako Corp. Anti-CD3 Pan T-conjugated and anti-CD11c antibody-conjugated magnetic beads were purchased from Miltenyi Biotec Inc. In some experiments, DCs at day 7–8 were incubated with Q Dots (Molecular Probes) for one hour (per the manufacturer instructions) and further allowed to mature as follows.

### Dendritic cell culture and activation

Bone marrow–derived dendritic cells (BMDCs) were prepared as previously described [38]. On day 7–8, loosely bound DCs were harvested by gentle pipetting and were activated with either TAA (40 μg/ml) or a pro-inflammatory cytokine cocktail (IL-1β, 25 ng/ml; TNF-α, 50 ng/ml; INF-α, 5 ng/ml; IFN-γ, 10 ng/ml; polyI:C, 10 μg/ml) or a combination of both (TAA and cytokines) for 18–24 hours. On day 8–9, activated DCs were harvested from the respective treatment group and subjected to downstream applications.

### Biomatrix entrapment of activated DCs

Various concentrations of fibrinogen (1–20 mg protein) and thrombin (0.1–25 International Units; IU) (Sigma-Aldrich), dissolved in sterile normal saline (NS), were mixed together and incubated at 37°C. Two milligram of fibrinogen protein was found to form an appropriate gel within 15–30 minutes. For the incorporation of cells into fibrin clots, desired numbers of DCs (1–10 × 10^5^) were pipetted into an Eppendorf tube and centrifuged (1200 rpm/5 min). The supernatant was removed completely under negative pressure and the cell pellet was suspended gently in 50 μl of fibrinogen protein solution (equivalent to 2 mg of fibrinogen protein). Care was taken to not introduce any air bubbles. Approximately 0.4 IU of thrombin protein was inoculated in a total volume of 2.5 μl. The preparation was incubated at 37°C for 15–30 min.

### Tumor induction and treatment using DC scaffolds in mice

For establishing primary tumors, TC-1 cells (80–90% confluent) were harvested using trypsin-EDTA. The cells were washed twice in phosphate buffered saline (PBS) (1200 rpm/RT/4 min) and ultimately suspended in PBS to a final concentration of 5 × 10^6^ cells/ml. Mice were anaesthetized and inoculated with TC-1 cells (5 × 10^5^/100 μl) at their shaved lateral flanks. Secondary tumors were established by a previously described partial resection method [39]. For some experiments, tumors were induced using TC-1 luciferase cells (5 × 10^5^/mouse). At appropriate time points, the luciferase activities were measured by inoculating mice intraperitoneally with luciferin substrate (1.8 μg/mouse). Fifteen minutes after the substrate inoculation, the luciferase activity was measured using an intravital imaging system (IVIS 100, Caliper Life Science) in total darkness. Also, tumors were induced using B16 melanoma cells in the same way as described above for TC-1 cells.

For treatment of primary tumors, DC scaffolds were placed subcutaneously on the same side as the tumor or on the contralateral side in anaesthetized animals. One set of mice (*n* = 4–5) that received a subcutaneous injection of free DCs or biomatrix without DCs was also included as a control. For the treatment of secondary tumors, at the time of surgical tumor resection, DC-carrying scaffolds were placed directly at the resected tumor site, and the wound was closed using 3–0 surgical sutures (Supplementary Figure S1A). The tumor burden in variously treated mice was determined using a Vernier caliper. At an appropriate time point, the mice were euthanized and various tissues (tumor, spleen and scaffolds) were harvested for histopathological, IHC, FACS, or immunofluorescence studies. In some assays, 3–4 weeks after treatment, splenocytes from variously treated mice (beDC in tumor bearing mice, beDC in normal mice and biomatrix in tumor bearing mice) were activated with phorbol myristate acetate (PMA; 10 ng/ml; Sigma) + ionomycin (1μg/ml; Sigma) for 12–15 hours. Golgistop (BD Biosciences) was added for the entire duration of incubation at 37°C in 5% CO_2_.

### Flow cytometry and cell migration assay

A phenotypic characterization of DCs and T cells was performed using the BD Accuri C6 FACS analyzer (Becton Dickinson). For FACS analysis, cells were suspended in freshly prepared FACS buffer (3% FBS in PBS) and stained with specific antibodies once the Fc receptor on cells was blocked using CD16/32 antibodies (15 min/4°C). Antibody-specific staining was performed for 30 min in dark/4°C. Stained cells were washed twice in FACS buffer and finally suspended in 1% paraformaldehyde (PFA) PBS. For intracellular staining of cytokines, Golgistop (BD Biosciences) was added 2 hours prior to cell harvesting and cells stained after permeabilization with BD Cytofix/Cytoperm^TM^. For cell migration assays, DCs or T cells were labeled with carboxyfluorescein succinimidyl ester (CFSE) (5 μM/10^6^cells). To evaluate the cell egression from biomatrix, DC scaffold or free DCs were placed in the upper chamber and allowed to migrate towards medium (negative control) or macrophage inflammatory protein (MIP-1α) in the lower chamber of a collagen-coated Transwell (CoStar, Corning, New York, USA).

To evaluate the migration of lymphocytes towards scaffolds, MACS-purified CD3^+^ T cells from tumor-bearing mice were placed in the upper chamber and allowed to migrate towards the DC scaffold or MIP-1α in the lower chamber for 48 hours. At the end of the study, the cells collected from the lower chamber were suspended in 300 μl of FACS buffer and were FACS analyzed by collecting events for 1 minute. For the analysis of DC-scaffolds under *in vivo* conditions, biomatrices were harvested from mice at desired time points. The cells were dislodged using a cell strainer (BD Falcon; 40 μm), appropriately stained, and analyzed by FACS. Stained cells were suspended in 1% PFA and kept dark at 4°C until analyzed.

### Preparation of tumor-associated antigen (TAA)

TC-1 cells were grown to full confluency with partial detachment. At the end of the incubation, cells were harvested using the TrypL (Gibco) treatment. Cells were washed and suspended in PBS. Harvested cells were subjected to five freeze-thaw cycles with in-between high-speed centrifugations (13000 rpm/10 min/4°C). Cell disruption was confirmed by trypan blue staining. The cell debris was removed by centrifugation, the supernatant was filter-sterilized (0.22 μm) and the protein concentration was estimated using the Bradford reagent (Bio-Rad). The prepared TAA was stored at −80°C.

### *Ex vivo* DC/splenocyte co-culture system

For the assessment of cellular interactions inside the biomatrices containing activated DCs, a co-culture system was developed wherein variously activated DCs and splenocytes from tumor-bearing mice were entrapped inside the biomatrix (DC:lymphocyte, 1:5) and incubated for 72 hours/37°C/5% CO_2_ submerged in cRPMI. The supernatant was harvested and stored at −80°C until analysis. At the end of the incubation, the cells were harvested and analyzed by FACS as described above.

The visual assessment of cellular interactions inside the biomatrix was carried out as described earlier [17]. In brief, DCs were labeled with Q Dots (Molecular Probes; per the manufacturer's instructions), whereas lymphocytes were labeled with CFSE (5 μM/10^6^ cells) and incorporated into the biomatrix. The whole structure was immobilized using 2.5% sterile agarose and covered with 200 μl cRPMI. Images were acquired using a live imaging system (Delta Vision DV Microscope, Applied Precision) at 20×. Images were captured every five minutes for 24 hours and analyzed using the SoftWorX ver 1.3 program. The cellular interactions and the velocity of moving lymphocytes inside the biomatrix were assessed using MatLab software (MathWorks^®^).

### *In vivo* homing assay and immunostaining

For homing assays, CFSE-labeled lymphocytes from tumor-bearing mice were injected intravenously (10^7^ cells/100 μl) through the tail vein into scaffold-implanted mice. The number of fluorescent cells recruited to various LNs (such as popliteal, inguinal and cervical), spleen and biomatrices were assessed using confocal microscopy 48 hours after the injection. Lymphoid organs and transplants were embedded in Tissue-Tek O.C.T. compound (Leica Microsystems) and were snap frozen in liquid nitrogen. Cryostat sections (5–7 mm thick) were cut and placed on an APS-coated glass slide (Fisher brand, Fisher Scientific). Sections were fixed with cold acetone for 5 min, dried, and kept at −80°C until used. After blocking with 0.1% bovine serum albumin (BSA) in TBS-T (Tris buffered saline with 0.05% Tween 20) for 1 hour at 20°C, sections were incubated for 1–2 hour at 20°C with appropriate antibodies diluted in the blocking buffer for each incubation step after three, 5-minute washes with PBS.

### Statistical analysis

Statistical analysis was performed using the unpaired Student's *t*-test (for homing assays and for the rolling and sticking fractions) or one-way ANOVA, as appropriate. Mice survival was assessed by the Log-rank test. GraphPad Prism6 and/or Microsoft Excel 2013 were used as appropriate. *P* < 0.05 was considered significant.

## SUPPLEMENTARY MATERIALS FIGURES AND MOVIES








