# Supplementary material for: Activated dendritic cells delivered in tissue compatible biomatrices induce in-situ anti-tumor CTL responses leading to tumor regression
Source: Oncotarget. 2016 May 20;7(26):39894–906. doi: 10.18632/oncotarget.9529 (PMC5129979; doi:10.18632/oncotarget.9529)
Supplement: Supplementary file 1 [file oncotarget-07-39894-s001.pdf]

Activated dendritic cells delivered in tissue compatible biomatrices induce *in-situ* anti-tumor CTL responses leading to tumor regression

Supplementary Materials

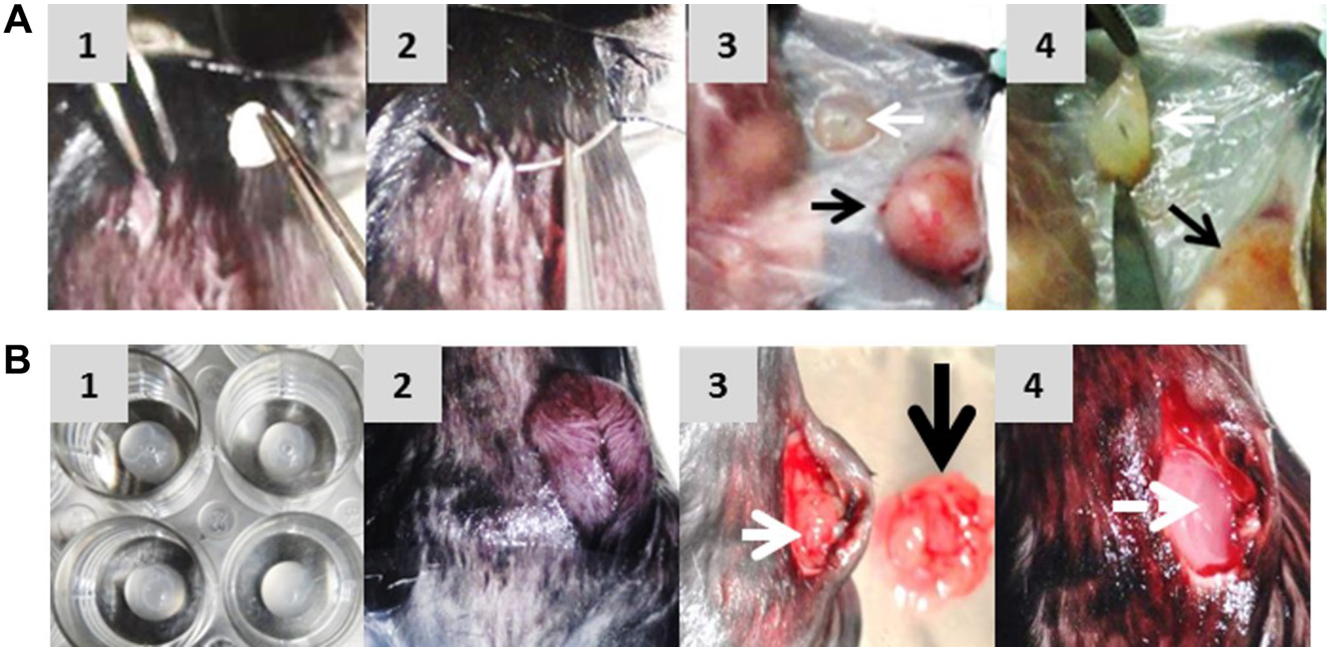

Supplementary Figure S1: Generation of DC scaffolds for tumor treatment. (**A**) Panel 1 shows the placement of DC scaffolds near the tumor site, panel 2 depicts the closing of the wound while panel 3 shows the physiological appearance of biomatrix 2–3 weeks post-implantation, and panel 4 shows the procedure of biomatrix harvesting along with host reactive tissue. White arrows show the biomatrix while black arrow shows the tumor mass. (**B**) Panel 1 shows the formation of flat DC scaffolds for the treatment of post-surgery secondary tumors. Panel 2 shows the tumor mass at the time of resection. Panel 3 shows the cavity created by the tumor resection (white arrow) and the removed tumor mass (black arrow). Panel 4 shows the placement of DC scaffold into the cavity for tumor treatment.

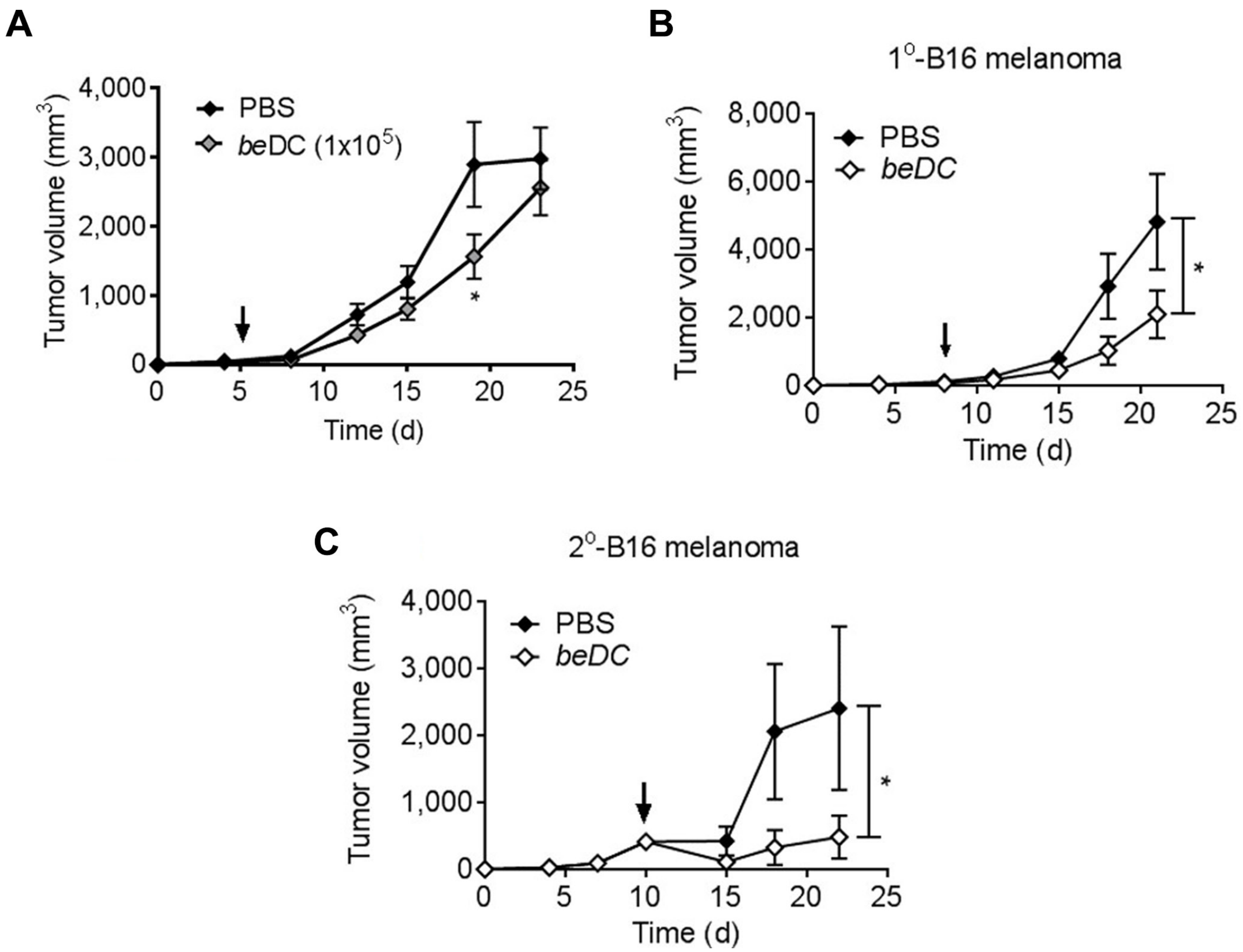

Supplementary Figure S2: (A) Treatment of TC1 tumors with biomatrix carrying 1×10^5 DCs. Treatment of B16 cell induced 1° (**B**) or 2° (**C**) melanoma using DC scaffolds. $n = 5–10$. Black arrow in A indicates the time point of commencement of therapy while black arrow in B represents the time point of tumor resection and DC-scaffold implantation. Data is represented as + SEM. *p-value < 0.05.

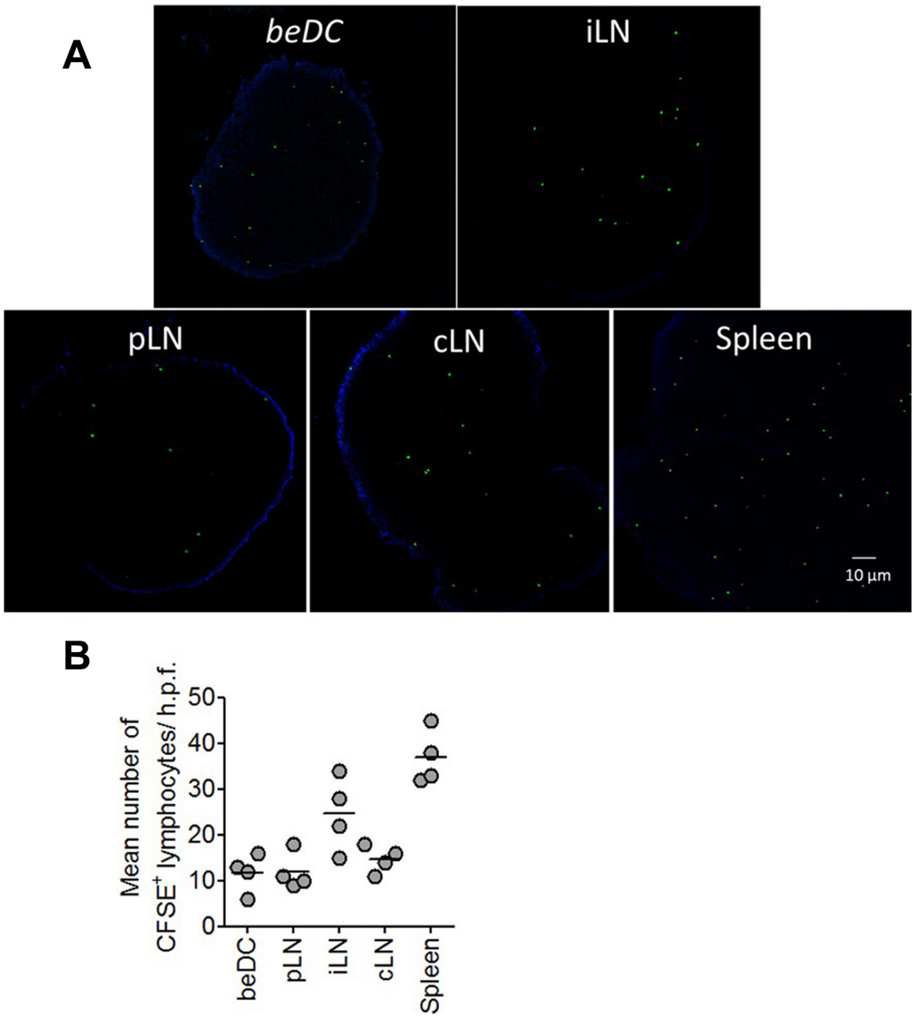

Supplementary Figure S3: Adoptively transferred lymphocytes reach host-implanted DC scaffolds with equal propensity compared with other SLOs. (**A**) Representative figures showing the relative movement of CFSE$^+$ lymphocytes into the biomatrix or SLOs 48 hours after adoptive transfer. iLN, inguinal lymph node; pLN, popliteal lymph node; cLN, cervical lymph node. (**B**) Quantification of infiltrating lymphocytes in high power fields.

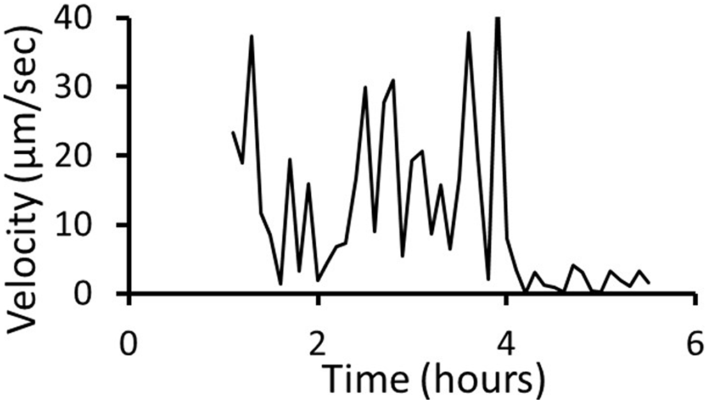

Supplementary Figure S4: Velocity of migrating lymphocytes inside the biomatrices under *in vitro* conditions. CFSE$^+$ lymphocytes and Q dot$^+$ DCs were put together in the scaffold and imaged using a live imaging system. The velocity was calculated using the MatLab® software.

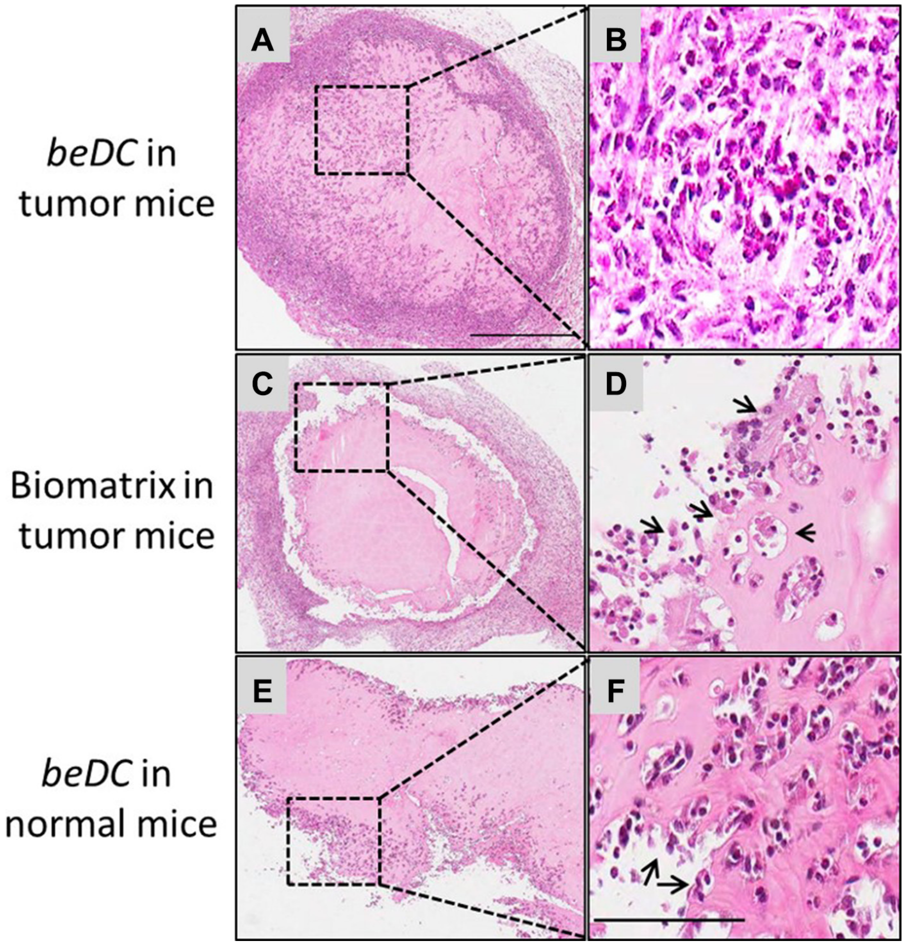

Supplementary Figure S5: Hematoxylin and eosin staining of biomatrices harvested from various treatment groups. (**A**, **C**, and **E**) are the 4x view of implants as depicted in the picture while (**B**, **D**) and (E) are the respective highlighted 40× areas. Black arrows in (**D** and **F**) show the sites of fibrin digestion by polymorphonuclear cells. Bar in A is equivalent to 1 mm while bar in (F) is equivalent to 100 μm.

Supplementary Movie S1: Interaction of DCs in biomatrix with infiltrating host lymphocytes. Though the DCs in scaffolds were stationary, the dendrites projected from them were making active physical contacts with infiltrating lymphocytes. See Supplementary_ Movie_S1

Supplementary Movie S2: Movement of infiltrated lymphocytes inside the biomatrix. Infiltrated host lymphocytes moved agilely and made intimate contacts with DCs for extended periods. See Supplementary_ Movie_S2

Supplementary Movie S3: Cell division of infiltrated lymphocytes in association with DCS inside the biomatrix. See Supplementary_ Movie_S3
